# UAS-Based Plant Phenotyping for Research and Breeding Applications

**DOI:** 10.34133/2021/9840192

**Published:** 2021-06-10

**Authors:** Wei Guo, Matthew E. Carroll, Arti Singh, Tyson L. Swetnam, Nirav Merchant, Soumik Sarkar, Asheesh K. Singh, Baskar Ganapathysubramanian

**Affiliations:** ^1^Graduate School of Agricultural and Life Sciences, The University of Tokyo, Japan; ^2^Department of Agronomy, Iowa State University, Ames, Iowa, USA; ^3^BIO5 Institute, University of Arizona, Tucson, USA; ^4^Data Science Institute, University of Arizona, Tucson, USA; ^5^Department of Mechanical Engineering, Iowa State University, Ames, Iowa, USA

## Abstract

Unmanned aircraft system (UAS) is a particularly powerful tool for plant phenotyping, due to reasonable cost of procurement and deployment, ease and flexibility for control and operation, ability to reconfigure sensor payloads to diversify sensing, and the ability to seamlessly fit into a larger connected phenotyping network. These advantages have expanded the use of UAS-based plant phenotyping approach in research and breeding applications. This paper reviews the state of the art in the deployment, collection, curation, storage, and analysis of data from UAS-based phenotyping platforms. We discuss pressing technical challenges, identify future trends in UAS-based phenotyping that the plant research community should be aware of, and pinpoint key plant science and agronomic questions that can be resolved with the next generation of UAS-based imaging modalities and associated data analysis pipelines. This review provides a broad account of the state of the art in UAS-based phenotyping to reduce the barrier to entry to plant science practitioners interested in deploying this imaging modality for phenotyping in plant breeding and research areas.

## 1. Introduction

Most air vehicles used for plant phenotyping are based on the concept of a remotely piloted aircraft system (RPAS) as defined by the International Civil Aviation Organization (ICAO). There are, however, a diversity of names and nomenclature for these devices depending on country of use, with drones, unmanned air vehicle (UAV), and unmanned aircraft system (UAS) being commonly used terms. In order to avoid ambiguity, we choose to call these systems as UAS, with the definition used by the United States Federal Aviation Administration (FAA): “an unmanned aircraft (an aircraft that is operated without the possibility of direct human intervention from within or on the aircraft) and associated elements (including communication links and the components that control the unmanned aircraft) that are required for the pilot in command to operate safely and efficiently in the national airspace system.” (Public Law 112-95, Section 331 (8-9) United States) [1].

There are several technical decisions that the practitioner has to make to ensure that the UAS operation and subsequent postprocessing analysis produce actionable information from the plant science perspective. The key decisions a practitioner needs to make include: Which UAV and sensor package should one choose? What are the critical steps to successful deployment, and steps to successful processing of the data? What has been done in this scientific discipline? What are current state-of-the-art applications of UAS in plant phenotyping? Where are we headed next? What are the open questions, trends, and challenges [2]? This paper reviews the state-of-the-art in UAS deployment, data collection, curation, storage, and analyses, discusses pressing technical challenges, and identifies future trends in this arena. The intent of this review paper is to provide an overview of the state of the art in aerial-based analytics to plant sciences and breeding practitioners who are interested in deploying this phenotyping modality matching their specific phenotyping needs. For complementary reading on UAS phenotyping topics not directly covered in our work, readers can refer to additional review articles [3, 4].

## 2. UAS Types and Imaging Modalities

### 2.1. Types and/or Classes of UAV

We first provide a taxonomy of UAV devices through the lens of plant phenotyping. While UAV can be classified based on a diverse set of features [4–7], in the context of a plant sciences/breeding practitioner, it is useful to simply classify them according to their physical features or configuration. UAV are classified into the following: single-rotor, multirotor, fixed-wing, and hybrid VTOL (vertical takeoff and landing) fixed-wing.  Table 1 provides a concise overview of these types of UAV. Prices are not listed, as it can vary substantially depending on the manufacturer and country that they are bought in; however, for comparison purposes, a price range is included with information from the US.

- *Single-rotor UAV* (also called helicopter) is a device that can be powered by either combustion (i.e., liquid fuel) or an electric motor, resulting in substantially longer flight times and higher payload. The earliest applications of UAV to plant phenotyping used these kinds of devices [8]. While providing reliability and flexibility along with larger payload capacity, the major disadvantage of such single-rotor unmanned helicopters remains their cost as well as the ensuing complexity of operation and maintenance
- *Multirotor UAVs* are currently the most popular UAV devices, primarily due to their ease of operation, low cost, and reasonable payloads. These devices have seen wide usage in a variety of applications including agricultural monitoring and industrial inspection. A major disadvantage of multirotor UAV is their limited endurance and speed, which creates difficulties for long runtime phenotyping. These limitations are a challenge in plant research where a large tract of field and experiments may need to be phenotyped. While this issue can be mitigated to an extent by the use of spare batteries, the problem requires considerations of battery energy density, weight, and cost. This is an active (and rapidly progressing) area of research [9] with several potential possibilities including (a) moving away from lithium ion batteries, (b) swapping UAV and wireless power transfer [10, 11], and (c) mobile charging stations [12]
- *Fixed-wing UAVs* provide an advantage over multirotor as these units can cover larger areas due to expanded flight time and speed; however, they require a “runway” for takeoff and landing. Additionally, this UAV type cannot hover over one spot, precluding detailed observations in specific cases where such functionality is needed, i.e., immobile measurement and tracking. The fixed-wing UAV can hold larger payloads, allowing multiple sensors to make simultaneous (and coregistered) measurements, thus increasing phenotyping capability. Fixed-wing UAVs generally fly at higher speeds than multirotor UAV, so some care has to be used to ensure that the capture rate of the sensor matches the UAV speed
- *Vertical takeoff and landing* (*VTOL*) *UAVs* are hybrid multirotor and fixed-wing UAV with capabilities to hover like a helicopter (for takeoff and landing, but not for phenotyping), high cruising speed, multifunctionality and versatility, and improved protection of sensors compared to fixed-wing UAV at takeoff and landing. Since this is a relatively new technology in civilian applications, the cost is prohibitive and the barrier to entry remains high for current practitioners in the plant science phenotyping

### 2.2. Open (Maker) vs. Commercial Types of UAS

A UAS system typically consists of the hardware (i.e., the actual physical system) and the control software (i.e., the programs that help run the hardware safely). With the advent of the maker movement, there are now two viable approaches to procuring a UAS. One approach is to buy an off-the-shelf UAS, while the other approach is to use open-source information to create and operate a UAS, where both hardware (via 3D printing) and control software (via open-source repositories) are starting to become available to prototype and deploy UAS that are tailored for a specific phenotyping application. For most beginning and intermediate users of UAS for plant phenotyping, especially for research and breeding applications, commercial UAS systems that provide an all-in-one package to rapidly sense, curate, and act on their field plots provide minimal barrier to entry. However, a clear understanding of the user needs and what the commercial UAS system can deliver is required for safe and hassle-free operation. Also, these systems are not generally customizable if such a need arises during the course of the experiments.

The primary technical difference between the two approaches deals with access to the control and command software. In commercial-type UAS, the flight control system is provided as proprietary software (usually as embedded firmware), which is integrated into the hardware. The end-user usually cannot access or make any changes to the source code. An important consequence of such control software code is the guarantee of performance and technical support during operation. In contrast, open-source flight control systems provide the source code to allow users to modify and integrate into their UAS. Most commercial manufacturers provide (finite time) guarantees of performance and also provide reasonable technical support to troubleshoot issues during phenotyping. In contrast, open-source codes are usually provided “*as is*”, with no expectation of performance or downstream technical support. Some examples of open source and paid for software for plot extraction and trait calculations can be found in the following references [13–15]. A GitHub repository associated with this paper for breeder-friendly UAS plant phenotyping can be found here [16].

### 2.3. Technical Considerations before UAS Deployment

There are multiple technical decisions that a practitioner must make to identify the most viable UAS for their specific application in plant phenotyping at research scale.

#### 2.3.1. Navigation and Geotagging

The control software needs accurate positioning to produce good geotagged data of the observed field. Geotagged data is essential for correlating genotype/management specifics in the field with the images/measurements made by the UAS. Most commercial UASs have dual global navigation satellite systems (GNSS) such as global positioning system (GPS) and GLONASS. These dual satellite navigation systems have an accuracy of about 2 meters (horizontal). This resolution may not be sufficient for some phenotyping applications, where accuracy in both horizontal and vertical direction in the 1-2 cm range is desired, and this can be achieved by integrating differential correction functions with the GNSS such as real-time kinematics (RTK). If the intent is to create an orthomosaic (stitched images) for the entire experiment or field, relative reference and GCP can be used without an RTK level accuracy. However, if individual images are analyzed and high resolution is required, RTK may be desirable.

#### 2.3.2. Weight vs. Battery

Increasing the weight of the UAS is useful for stability (reduced buffeting with wind shear) as well as improved payload carrying capacity. However, increased weight substantially reduces the total flight time of a UAS due to rapidly draining battery as more power is required to keep the UAS afloat and for maneuvering. Most current commercial multimotor UAS can only fly up to 30 minutes, depending on the sensor payload, which may not be enough to cover large experiments/fields. Therefore, if flying large experiment fields, batteries are swapped between flights and/or multiple UASs are operated in tandem. For smaller sized programs, this is not an important constraint.

#### 2.3.3. Multiple UAS Operating in Tandem

UAS-based imaging can enable the 3D reconstruction/mapping of the complete experiment/field, because images of a single location are taken from different perspectives allowing 3D reconstruction using *structure from motion* (SfM) [17]. However, unless imaging from multiple perspectives is done very rapidly, the effect of wind can be fairly significant in reshaping the canopy (effectively changing features via occlusion, bending, etc.). One way to circumvent this challenge is to take multiview images simultaneously, which can be accomplished by operating multiple UAS in tandem. We foresee several promising tools and frameworks becoming available to the plant science community that could take advantage of higher quality 3D point clouds that are generated from deploying multiple UAS in tandem [18–23].

#### 2.3.4. Policy Challenges Using UAS

There is no standard policy for operating UAS, with variations even within each country. This is understandable as the UAS ecosystem is rapidly evolving. It is important for a scientist/breeder to check and conform to both national and local regulations before deploying the UAS [24–26].

### 2.4. UAS-Based Imaging Modalities

**Figure 1:**
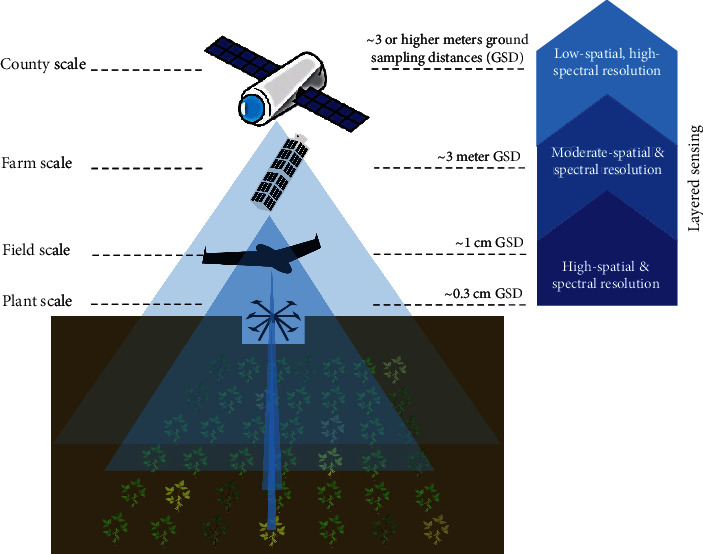
UAS across phenotyping scales, sensing levels, and ground sampling distance (GSD). Image is for illustration purposes and not to scale.

Aerial imaging includes plant, field, farm, and country scales using different systems from drones to satellites (Figure 1). For this article, we primarily focus on plant and field scales.

#### 2.4.1. RGB Digital Camera

The most commonly used imaging system in UAS is an RGB (red, green, and blue) digital camera. They are particularly attractive due to their low cost, low weight, and high resolution. Additionally, due to their similarity to the electromagnetic spectrum over which the human eye operates, RGB camera-based UAS image data has been successfully used for automated phenotyping of features that have traditionally been manually performed. Examples of morphological traits include height, leaf area, shape, organ detection and counting, plant density estimation, and plant/weeds discrimination, among others [27–45]. Most popular UAS systems are integrated with a RGB camera system, thus allowing real-time image preview, seamless camera configuration management, and simple remote trigger control by the operator. Due to the tight hardware integration with the UAS system, the RGB images collected are geotagged with onboard GPS data. This minimizes subsequent downstream problems with georegistration.

#### 2.4.2. Multispectral Camera

Cameras that can image at a small number (usually between 3 and 10) of wavebands of the electromagnetic spectrum are called multispectral cameras. From the plant science perspective, cameras that measure red, green, and blue bands, along with measurements of the near-infrared and red edge bands, have been widely used. This is because the reflectance of chloroplast has a peak in the near-infrared band (around 850 *μ*m) and changes rapidly at the red edge (around 700 *μ*m) band. Thus, by combining these bands, one can measure various vegetation indices [46, 47]. More recently, multispectral cameras with dynamically selectable bands have become available. These systems are particularly promising for capturing different phenotypes that exhibit differing signatures at different wavelengths. Recent work has shown that carefully selected multispectral bands in conjunction with sophisticated machine learning (ML) tools can result in sensitive approaches to early detection of a variety of plant traits, including stress signatures [48].

#### 2.4.3. Hyperspectral (HS) Camera

Cameras that can image across a large number of wavebands of the electromagnetic spectrum are called hyperspectral cameras. Hyperspectral cameras have traditionally been used at two scales: (a) on the single plant scale or (b) at the field scale. HS cameras provide significant advantages over other imaging modalities due to a wider electromagnetic spectrum coverage enabling more diverse trait measurements. HS camera can provide physiologically meaningful information about the biophysical and biochemical properties of crop species, as well as detection of biotic and abiotic stresses [49, 50]. A recent development in HS cameras includes the commercialization of “snapshot” HS cameras where all bands are simultaneously captured; however, it is a developing technology in plant science applications. The availability of HS cameras that can be reliably deploy onto a UAS is expected to complement high-throughput phenotyping, as they have the capability of not only providing HS information, but potentially can be used to create 3D point cloud data across each registered spectral band. However, the current challenges to deploying HS camera payloads include (a) low spatial resolution, or rather low spatial-spectral resolution trade-off; (b) high power requirements; (c) calibration, especially for field deployment under varying illuminations; and (d) downstream data analytics to extract useful traits. These are areas of very active research, with viable solutions on the horizon [51–57].

#### 2.4.4. Thermal Camera

Thermographic imaging measures the infrared part of the electromagnetic spectrum from an object. This is physiologically important because healthy plants (specifically leaves) emit radiation in the infrared part of the spectrum. Various abiotic and biotic stresses can be indirectly related to the infrared emission signature of the canopy. This is because stresses (heat, drought, and biotic) can result in altered rates of photosynthesis and transpiration, thus affecting the canopy temperature and hence the thermal signature. Therefore, thermal imaging can be a high-throughput approach to evaluating the physiological state of the plant. However, deploying thermal cameras on UAS has seen limited usage due to difficulties including hardware integration, cost of the camera, low frame rate capture, and resolution compared to RGB cameras. Additionally, the thermal image of the field is influenced by the surroundings (presence of roads, water bodies, and buildings) and thus requires calibration. As a consequence, the use of thermal cameras deployed on UAS has seen fewer successful applications in field-based plant phenotyping than RGB imaging [58–61].

#### 2.4.5. LiDAR (Light Detection and Ranging)

Although earlier use of LiDAR-based systems used planes or ground-based systems, the reduction in size and weight of LiDAR instruments makes it usable on UAS with appropriate data analytics pipelines. Since LiDAR uses lasers to create dense 3D point clouds, it can provide a more detailed information than what is achievable from SfM or other methods using regular digital or multispectral cameras [62]. Furthermore, LiDAR is amenable for time series tracking of object or plant organ geometries [63]. UAS-mounted LiDAR-based phenotyping has been used for the estimation of canopy biomass and plant height, for example, canopy height in winter wheat to the effect on nitrogen fertilizer rates [64], sugarcane biomass estimation [65], and maize height tracking in lodged plots [66]. The current challenges with routine utilization of LiDAR on UAS are the cost vs. quality trade-off of data [67]. Additional challenges include data processing standardization and the large size of the data. LiDAR is still an emerging technology for use on UAS, and with further research, its usefulness may increase to phenotype additional traits. An in-depth review of LiDAR for plant phenotyping uses was provided by [68].

Table 2 lays out the main types of sensors used as UAV payload. The cost, weight, resolution, and ease of use are presented in categories rather than numbers, because there are a wide range of sensors within each category with varying parameters.

### 2.5. Open Technical Challenges with Payload Integration

A promising approach in recent high-throughput phenotyping experiments has been to simultaneously deploy multiple imaging modalities. The motivation here is to simultaneously extract complementary traits using different modalities (RGB+thermal, for instance). However, there are significant technical challenges, e.g., coregistering and combined image analysis, that have to be resolved before this becomes the standard. Challenges span the range from deployment to analysis and include (i) remote and simultaneous triggering of multiple separately mounted imaging systems, (ii) geotagging multiple image data streams, (iii) image coregistering/alignment between cameras and between bands, and (iv) mismatch in image resolution across cameras and associated signal-to-noise ratios. Resolutions to these challenges are active areas of research [69–71]; for example, these include the use of structure from motion (SfM) tools to create georeferenced orthomosaic image for each band followed by overlaying of distinct band information based on the geoinformation. A maintained list of platform agnostic SfM software is available at [72].

## 3. Preprocessing and Data Preparation

### 3.1. Ground Control Points (GCP)

Deploying UAS usually involves flight planning to ensure that data generated can be registered. The key steps involve the preparation and placement of ground control points (GCP) and way point selection. Ground control points are the visible marked targets placed on the surface of the observation field that are used to geocalibrate the UAS-based images. These targets are placed at locations that are premeasured by high precision GNSS (e.g., RTK-GNSS) and are associated with high-precision coordinates. Availability of these GCPs greatly increases the geometric accuracy of UAS-based mapping [38, 73–80]. Specifically, the presence of GCPs provides the capability to correct the latitude and longitude of all points (i.e., all collected images) to accurate GPS coordinates. This is critical to subsequently associate extracted traits with plot level information (for instance, locating and curating data across microplots from different observation dates).

#### 3.1.1. GCP Types

As visual targets that must be easily captured by the onboard imaging systems, GCPs must ideally (a) be clear and visible from the heights the UAS is being deployed and (b) have precisely measured GPS coordinates. There is no set standard for GCPs; however, the most common GCPs include rigid boards painted with an “X” shape marker, a checkerboard texture, or a circular target with a center marking. There are broadly two approaches to deploying GCPs for UAS deployment—temporary versus permanent. In the temporary approach, one places and calibrates the GCPs for each UAS flight campaign. The advantage of this approach is that there are no concerns about the material quality and robustness of the GCP, but the disadvantage is the time and effort needed to place and calibrate GCPs for every flight survey. Additionally, one can potentially change the location of the GCPs for every flight survey according to development stage and imaging conditions (for example, for pre- versus postcanopy closure). In contrast, in the permanent approach, the GCPs are fixed for the entire growing season. The advantage here is that the GCP placement is a one-time resource investment. Care has to be taken to identify locations of GCP placement so as not to hinder crop management practices while providing visual access to GCPs across the growing season. Additionally, the GCPs must be robust enough to withstand natural weather variability. Finally, there are emerging technological solutions that provide built-in high-precision GPS capability within each GCP [81]. High-precision and easy-to-use smart GCPs with built-in GPS may become more common in the near future.

#### 3.1.2. GCP Placements

The number and spatial distribution of GCPs affect the accuracy of mapping of the image data. Thus, increasing the number of GCPs and evenly distributing them over the imaging area is a possibility. However, as described earlier, obtaining good GCPs for large fields can be time-consuming and laborious. There are several recent studies that seek to identify the optimal number and spatial distribution of GCP placement [73, 78, 79]. For plant breeding applications that demand accurate extraction of microplots via high quality 3D mapping, at least 5 GCPs may suffice with four of them located at each corner and one located in the center of the observation field [79]. In plant breeding application, one GCP for every 200 m^2^ is generally appropriate. If a practitioner is trying to determine the optimum number of GCPs, they can refer to [77], and the GUI developed for easy use [82]. There are other options to GCPs, and we leave it to the practitioner to decide which method works best for them and fits within their budget. The uses of ground control points (GCPs) vs. real-time kitematic (RTK) vs. postprocessed kinematic (PPK) are common techniques for generating accurate UAS data products.

### 3.2. Way Point Selection and Placement

It is usually ideal to break up the flight path of the UAS into distinct “legs” of traversal (flight), with clearly identified start and end points in space (locations and height). These points are called *way points*, and the strategy of the UAS following a sequence of way points is called way point routing. Among other advantages, such way point routing ensures that the flight mission is repeatable, safe, and accurate. The predesigned way points record the GPS and inertial measurement unit (IMU) data, as well as camera action commands; thus, ensuring that the UAS follows the predesigned flight automatically. There are various software tools available for way pointing that abstract out the complexities via easy-to-use graphical user interfaces. The software is able to generate these points by the user entering the camera parameters, such as focal length and sensor width, and then inputting the flight altitude or desired GSD. A partial list of such software is listed in Table 3.

Some practical considerations while selecting way points include considerations of the desired spatial resolution and quality of the 3D mapping. The spatial resolution is related to the flight altitude and camera characteristics and must be carefully considered for individual phenotyping exercises. For educational purposes, given the flight altitude *A* [m], camera CMOS size *L* [m], corresponding pixel number *N* [pixel], and focal length of camera *F* [m], we can calculate spatial resolution *R* [*m*/pixel] as *R* ≈ (*A* × *L*)/(*F* × *N*). The quality of the 3D mapping requires that the images captured by the UAS enjoy high overlaps between images [73, 77, 98–100]. However, higher overlap increases the flight duration significantly thus limiting coverage. For dense vegetation and fields, it is recommended to have at least 85% frontal and 70% side overlap for ensuring good 3D mapping [101]. For easy-to-use calculations and estimations of flight time, we refer to the online mission planner tool [102].

### 3.3. Calibration

#### 3.3.1. Color Calibration: Approaches and Challenges

Most popular UAS with built-in imaging unit comes with an RGB color camera, although researchers also use specialized cameras with triband including the near infrared, particularly when estimating vegetation indices. While RGB cameras provide high-resolution images of the observation fields, variation in illumination as well as differences in camera hardware can result in the same scene being captured with slightly different colors. This calls for color calibration, which is a process of adjusting the pixel color values in images to a consistent value. Color calibration is especially important if the phenotype of interest is evaluated based on color. This is the case for most plant stress detection and quantification; for example, iron deficiency chlorosis (IDC) in soybean evaluation of symptoms is based on the extent of chlorosis (yellowing) and necrosis (browning) [103, 104]. Additionally, any comparative assessment between images from multiple UAS imaging times requires color calibration.

In field imaging via UAS, there are several factors that affect pixel data including illumination intensity, angle of the incoming light resource, spectral reflectance of the objects, relative position of the camera to the objects, and camera optical characteristics [105]. A common color calibration approach is to place a physical color calibration chart in the field, so that the UAS can concurrently collect data while imaging the calibration chart. This allows postflight standardization of the images based on the image characteristics of the color chart captured by the UAS [106]. However, even with the color calibration, care has to be taken into account for camera-specific variabilities (such as gamma correction [107–109]). Another physical constraint is that not every aerial shot can contain the calibration chart. A common assumption made is that the imaging configuration remains constant for the period that the aerial shots do not include the calibration chart. In this situation, the RGB digital cameras deployed on UAS can be used to extract morphological traits like height, shape, area, and counts instead of-color related traits that require parsing out subtle differences between genotypes.

#### 3.3.2. Spectra Calibration: Approaches and Challenges

When using multi- or hyperspectral cameras on UAS, sensor calibration is usually carried out to ensure that each pixel faithfully captures the data across the full spectral bands, thus producing reliable reflectance datacubes. In general, for agricultural research and breeding applications, calibrated reflectance datacubes provide sufficient information for subsequent physiologically meaningful analysis and decision support. Calibration of the camera is a complicated procedure that is usually taken care of by the manufacturer; see [110]. The conversion of the calibrated camera recordings to reflectance values is usually performed by using reflectance reference targets on the field. These reflectance targets have known spectral reflectance and are used to transform the camera readings into calibrated reflectance values [4, 111–115]. The standard approach to process this data is called the empirical line method (ELM). Recent work has suggested that ELM-based reflectance computing is suitable for flights under 30 minutes with stable weather conditions [116]. Care has to be taken to ensure that no significant illumination changes occur within each flight.

### 3.4. Software and Cyberinfrastructure

**Figure 2:**
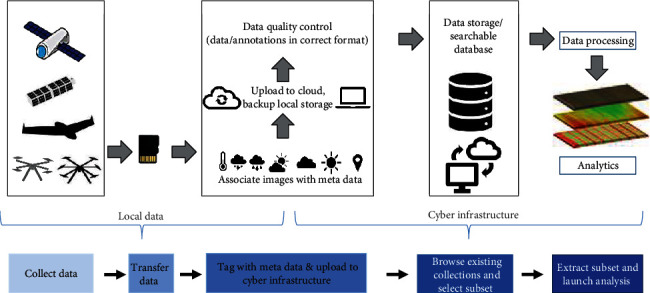
UAS workflow pipeline: data collection, transfer, upload, storage, and analytics.

UAS campaigns can amass large amounts of data fairly quickly. Therefore, having a well-defined data management strategy that facilitates multiple analysis workflows and subsequent integration of output data with decision support systems is essential (Figure 2).

The landscape of service providers that offer turnkey solutions is evolving rapidly (Table 4); at the same time, academic groups are producing ready-to-use open-source analysis workflows powered by deep learning methods [117]. Having a responsive cyberinfrastructure that can effectively leverage both commercial and academic offerings, while scaling (up and down) as the needs of the project evolve is paramount. Supported research cyberinfrastructures (in the US), like NSF CyVerse [118], XSEDE [119], and OpenScienceGrid [120], support the processing and hosting of nationally funded US-based research. Commercial cloud-based turnkey solutions for UAS data management, analysis, and team-based collaboration provide easy-to-use integrated viewers, applications, and app stores (Table 4). Many of these offerings have limits on allowable storage per tier and may not be ideal for a large long-term archival storage. Commercial cloud providers (for example, AWS, Google, and Azure) provide services for managing data through tiered storage and lifecycle management (highly redundant to slower long-term archival). This allows data to migrate from various tiers in an automated and cost-effective manner, and these capabilities can complement local IT resources, when feasible [121–123]. However, institutions may have restrictions on the use of some services and platforms, and this needs to be determined at the planning stage of experiments.

#### 3.4.1. Software

UAS-related scientific software can be broken down into categories: (a) UAS flight control and sensor orchestration (see earlier section), (b) passive sensor (i.e., imagery) image processing and analysis, (c) active sensor (i.e., LiDAR) processing and analysis, (d) statistical and analytical GIS, and (e) data management and collaboration. In general, financially expensive solutions involve complete integration of the UAS, sensors, and analytical image analysis pipelines via cloud processing services. These software can be open-source or commercial. Open-source software solutions are more granular, offering components of the UAS analysis pipeline with varying levels of integration and interoperability.

*(1) Open-Source Software*. The OpenDroneMap (ODM, [124]) project supports an open “ecosystem of solutions for collecting, processing, analyzing and displaying aerial data; and to build strong, self-sustaining communities around them.” OpenDroneMap includes a stand-alone program, web interface, API, and connectivity to multinode cloud processing options. ODM data can be uploaded to the OpenAerialMap.

*(2) Commercial Software*. The UAS surveying industry for civil infrastructure is the most lucrative and largest sector for software development. Many software are packaged as part of UAS surveying ecosystems (Table 4). Example solutions include SenseFly ([125]) and ESRI Drone2Map. These have partnered with Pix4D (Pix4Dfields, [126]) and DroneDeploy, respectively. Other example software for image processing and SfM with multiview stereo (SfM-MVS) photogrammetry includes Agisoft Metashape. Most commercial software (e.g., Pix4D and Agisoft) can be run on bare metal or cloud infrastructure, in single-node or multinode configurations.

#### 3.4.2. Database Management Strategies

UAS campaign data is typically acquired on removable flash-based memory cards and often transferred to field-based laptops that are synchronized to more permanent storage resources such as file servers and cloud. Maintaining a catalog that allows locating of files that are offline (on cards or USB drives) or across multiple systems is essential. Cataloging software can be used to keep track of data distributed across different storage media. Examples include abeMeda [140] and NeoFinder [141]. Cataloguing software can be coupled with cloud backup software to provide recovery, if needed.

Common UAS data file types include orthomosaic rasters (e.g., tiff, geotiff, HDF5, and NetCDF) of spectral indices, as well as dense point clouds (e.g., las, laz, bil, and ply). UAS datasets are highly heterogeneous and epitomize the “long tail” of research data. Unstructured data are typically the largest and also the least informative. Unstructured data, stored on local hard disks or in cloud-based object storage (buckets), have significant input-output (IO) requirements, which make moving, reading, or writing of large datasets slow and impractical at scale. Critically, UAS data are also at risk of becoming “dark data” [142]—either lost or becoming unusable by the rest of the science community. In order to make large quantities of data more available for analyses, these data need to be given structure in the form of an index. Structured indices, e.g., PostgreSQL with PostGIS extension [143], MongoDB [144, 145], and ElasticSearch (based on Apache Lucene) [146], allow rapid search and query of UAS data. Indexing of UAS data is critical to its findability, accessibility, and reuse. However, these require dedicated cyberinfrastructure hardware for hosting of indices and technical expertise. Recent work has worked on extracting traits from images, while reducing data size and storage needs [147].

Enterprise processing software (e.g., ESRI, Pix4D, and Agisoft) offer cloud storage at additional cost. OpenAerialMap provides hosting for limited extents. Cloud-based providers, e.g., DroneDeploy and FarmersEdge, offer enterprise solutions for raw image and orthomosaic data management. These solutions are most likely the easiest to use for novice UAS operators, but more expensive than hosting own services at scale for a mid- to large-scale research effort, e.g., a regional research laboratory or national research branch. Research needs differ from commercial solutions in several distinct ways, including the need to maintain and to curate data (often in perpetuity), and to provide provenance and sharing to ensure findable, accessible, interoperable, reusable (FAIR) data principles are met [148, 149].

#### 3.4.3. Data Sharing and FAIR Principles

While collecting UAS-based data is important, extracting actionable scientific insight calls for good data curation, storage, sharing, and reuse [150]. This is especially true if substantial resources are expended in collecting large quantities of UAS-based imaging data, which can be used by multiple groups to answer complementary research questions. This requires adhering to metadata standards that are consistent with community-established needs. We encourage practitioners to consider reviewing best practices from the Open Geospatial Consortium (OGC) unmanned systems working group [151], as well as others, e.g., Sensor, Observation, Sample, and Actuator (SOSA) ontology [152] and dronetology [153].

#### 3.4.4. Integration with External Systems and Extensibility

Analysis pipelines and workflows for UAS data range from “intricate” to “bespoke” by virtue of their specific use cases, number of steps required for processing, and preferred software. It is fairly common to exceed the computational resources available on a single server or workstation as the amount of data increases. Solutions require incorporation of workflow management systems (WMS) that support the ability to distribute tasks among distributed (external) computational resources (clouds, HPC, etc.) and manage the execution and recovery from failures while processing large volumes of data. WMS also afford the necessary reproducibility [154], by keeping track of input parameters used for applications and processed outputs for every step, with the ability to perform advanced analysis that requires parameters sweep, e.g., building models for ML applications. Example methods for reproducibility include the use of SDKs and APIs such as the Pix4DEngine, Agisoft Metashape Python or Java pipeline, and the OpenDroneMap ecosystem. Examples of WMS systems include ArcGIS workflow manager, Dask [155], Makeflow, and WorkQueue [155, 156].

Data derived from UAS analysis are often shared with stakeholders and users not conversant with UAS data products. The ability to rapidly review, iterate, and share data products, as well as gather and track user feedback, is important to improve data management. Use of online web services for data visualization can help to increase the speed at which teams can share and use data with tools like GoogleMaps API, QGIS Web Client Survey, and ArcGIS Online. Use of productivity applications for task management (e.g., Trello), source code repositories (e.g., GitHub), documentation (e.g., Atlassian Wiki, Read the Docs), and concurrent document editor (e.g., Overleaf and Google Docs) is central to ensuring the required productivity in groups with varied levels of expertise and familiarity. While many commercial turnkey solutions provide these capabilities as part of their integrated platform, utilizing a good data and analysis management strategy will allow the inclusion of more applications in any analysis pipeline through use of URI, webhooks, and API calls provided by each of these applications.

## 4. UAS-Based Imaging of Plant Traits

**Figure 3:**
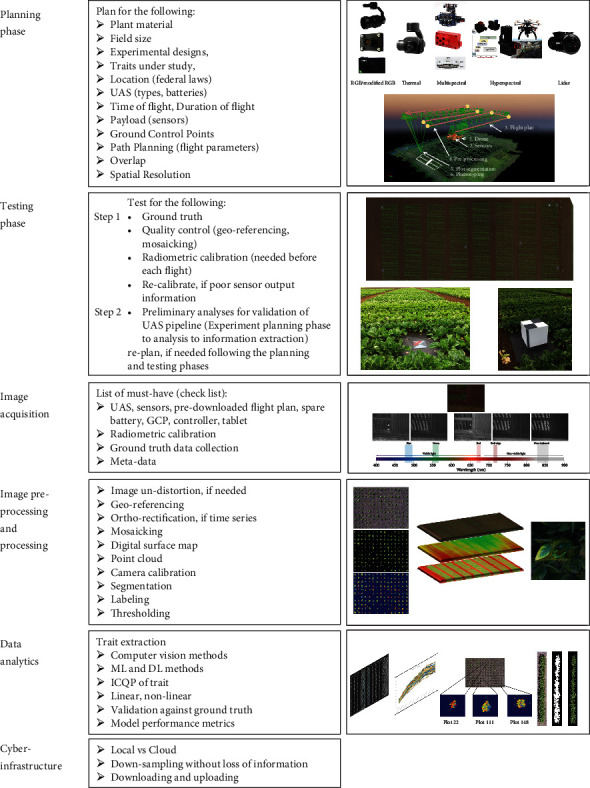
Establishing and conducting UAS-based experiments requires the establishment of an integrated pipeline with these stages: planning, testing, image acquisition, image preprocessing, image processing, data analytics, and cyber infrastructure. In this schematic, major considerations for each of these phases are described along with visuals for each phase. Readers can visit the wiki page [16], which is kept updated with the core techniques, pipeline, and source code related to UAS-based plant phenotyping.

Combination of spectral wavebands and other predictor traits with ML-based analytics has shown utility in crop yield and physiological trait measurement and prediction [157, 158]. Similarly, integration of crop, genetic, and weather parameters shows usefulness in crop yield prediction using deep learning [159]. Also, ground robot-based organ level phenotyping in soybean has also shown success in field conditions [160]. These are just a few examples of the value of involving UAS-based phenotyping to increase the scale of phenotyping for improving crop yield. Broadly speaking, UAS-based remote sensing can be used to phenotype numerous traits, including (i) performance traits such as yield and its components, canopy biomass, growth and development, and physiological and morphological; (ii) plant health traits such as abiotic and biotic stresses; and (iii) chemistry: sugar, proteins, metabolites, and high-value chemicals.  Figure 3 provides a schematic outline of the entire UAS-based pipeline that enables generation of plant trait information for breeding and research, as well as crop production applications. In Table 5 we cover recent literature with a focus on performance and plant stress traits; however, it must be noted that chemistry traits are also amenable with UAS phenotyping, although literature is sparse on the use of UAS for metabolites and chemicals phenotyping (see for example, [161]). More information specific to plant stress digital phenotyping can be found here [162–164].

While the majority of these studies used higher flight altitude (>25 m), the UAS types used are predominantly multirotor and utilize a combination of non-ML approaches for analysis. The use of multirotor in more recent literature could be due to a study bias as these papers are research experiments. Due to the constraints of payload weight and battery drain, it is likely that in precision and digital agriculture applications, fixed-wings and high altitude UAS will be desirable to cover large tracts of land with trait-dependent pixel resolution and/or complemented with significant advancements in sensor hardware. Due to the focus of this review on research and breeding applications, we do not delve deeper into precision and digital agriculture applications; however, the principles broadly remain consistent. Due to the continual push on image-based phenotyping in research, breeding and digital agriculture, pixels will continue to become more important, as future research may attempt to achieve greater information per unit pixel that comes from more trait estimation and better granularity.

ML methods have been successfully utilized at multiple scales, for example, microscopic level for nematode egg count [165], organ or object detection in canopy [160, 163, 166] or roots [167–170], yield prediction [157–159], disease identification and quantification [48, 49], and abiotic stress identification and quantification [103, 104]. Tools are also being developed for plant scientists to reduce the barrier to entry for ML utilization for plant phenotyping tasks [171]. With the robust set of examples where ML has been successfully used in crop trait phenotyping with ground-based systems, the transferability to UAS-based phenotyping and trait information extraction should be less cumbersome.

## 5. Key Trends and Outstanding Challenges

UAS-based phenotyping systems provide many attractive features to advance crop breeding and research. These include simultaneous phenotyping of multiple traits, assessment of larger genetic panels, mapping more complex traits including canopy shape, rapid phenotyping saving time and resources, time series data collection, and improved accuracy of measurement. With the current software and platforms, the barrier to entry has been significantly reduced. In this review article, we covered deployment, data collection, curation, and storage, while not focusing on data analytics since this has been covered in other papers. Advanced data analytics, such as machine learning, and particularly deep learning approaches have transformed the field of UAS-based applications in multiple domains including plant sciences, as it allows extracting complex, nonlinear, and hierarchical features from multiple sensors, including but not limited to digital, multi-, and hyperspectral cameras. Machine learning for plant phenotyping has been covered previously in review articles [117, 162, 206].

We conclude this review by identifying three broad classes of challenges that currently bottleneck increased and diverse use of UAS for plant phenotyping:

### 5.1. Challenges Associated with *Information Constraints*

The amount of useful information that can be extracted from the UAS payload determines the utility of the phenotyping exercise. Some of the pressing challenges associated with extracting viable information from UAS payloads include:

- *Low resolution*: UASs have lower resolution when compared to ground-based digital phenotyping campaigns. Especially with multispectral and hyperspectral imaging, the (spatial and spectral) lower resolution of UAS limits extracting fine-scale features at the individual plant scale. Promising approaches will rely on concepts of spatial- and spectral- superresolution, as well as PAN sharpening. Ongoing research seeks to obtain more information per pixel using these strategies [207–209], which will enable more traits to be estimated with better granularity. We envision that superresolution and PAN sharpening analysis will become more prominent as it attempts to infer subpixel information from data and maps between low- and high-resolution images collected from different UASs. These developments will also advance remote sensing capabilities to provide proximal level sensing including with smartphones [104]
- *Coregistering multiple sensors*: complex traits can be extracted if multiple sensors (thermal, RGB, multispectral) measure the same object. However, with sensors exhibiting different measurement frequencies as well as spatial resolution, accurately coregistering the sensor stream is an important prerequisite for viable trait extraction. Physical infield controls, and ML-based semantic segmentation and registration tools will be needed to perform seamless coregistration of data coming from different sensors. This also creates further complexity in data fusion for real time *in situ* processing as well as offline, deferred analytics. While not necessarily a constraint of UAS, this is an important factor for downstream image analysis for trait extraction and coregistering
- *Standardizing methods for complex trait extraction*: a persistent challenge remains our (lack of) ability to evaluate protocols for trait extraction without very resource intensive ground truthing. This is especially true for the conversion of 2D images into 3D point clouds. For instance, a presumably simple trait like canopy height remains a challenge. There is (not yet) a standard approach to height calculation based on SfM [28, 30, 36, 40, 210, 211], which is due to issues of wind, quality of 3D point reconstruction, and lack of consistent approaches to evaluating developed techniques. This issue is exacerbated for more complex canopy traits (especially time series data) due to wind effects and occlusion, as well complex plant organs. Recent approaches to overcome this challenge are the use of LiDAR in conjunction to SfM. Also, coupling of ground robotic systems [212] with UAS may be desirable to phenotype traits obscured from the UAS
- *Automated Plot Segmentation and Labeling*: another active area of research is plot segmentation with minimal previous work on automatic microplot segmentation using UAS data. Generally, a polygon of each plot is drawn manually or semiautomatically using GIS-based software such as QGIS or ArcGIS [30, 174, 210]; therefore, a fully automated solution is desirable especially in a breeding program that involves thousands to hundreds of thousands plots [14]
- *ML and DL problem*: ML and DL methods for plant phenotyping are an active area of research, and we suggest readers who are interested in this analysis refer to [162, 164, 206] as a starting point. While ML and DL are useful tools for UAS phenotyping, care needs to be taken to ensure that the data and problems trying to be solved are compatible with these methods (this includes large data size and variability). An appropriate choice of supervised or unsupervised ML methods is also crucial. In supervised learning, large labeling sets are needed, and in such cases, active learning may be useful [213]

### 5.2. Challenges Associated with *Power Constraints*

Due to current battery power limitation of UASs, large fields cannot be phenotyped efficiently. Current solution for covering a large field is to change the battery frequently, but it requires increased investment in batteries, and additionally, opens up issues of consistency caused by reboot of on board sensors. Several potential approaches are being explored to circumvent this constraint.

- These include (i) *on board* energy harvesting to extend the flight capacity [10, 11], (ii) *in situ* processing to reduce the storage requirements [214], and (iii) environment aware flight planning to maximize the time the UAS can stay afloat [77]. Additionally, mobile charging stations built on solar and other renewable energy sources have the potential to overcome the power constraints and increase operational flexibility
- Development of new sensors that integrate multiple capabilities along with improved GPS systems is also needed. As battery efficiency continually improves, sensors and on-board processing units with reduced energy demand are needed to overcome the hardware constraint
- Another promising option is via swarm UAS systems [215]. Swarm UAS systems are systems where multiple UAS autonomously traverse the field, collect data, perform data fusion (from multiple sensors), and provide improved overlap, and hence, increased area coverage [216]. However, regulation currently prevents UAS flights from swarming in an autonomous manner in many countries, including the USA. In this context, we note that recently Oklahoma State University received an approval for one pilot to operate multiple UASs in national space

### 5.3. Challenges Associated with *Policy Constraints*: UAS Operation Certification and Policy Advances

As the application of UAS is rapidly gaining prominence in multiple disciplines, there is a need for a cohesive voice from practitioners to help shape policies around certification and utilizations. For example, flights near restricted spaces can be a challenge for production or research fields in the vicinity of such restricted spaces. Additionally, there are limitations on UAS usage such as delivery of crop protection products in commercial fields. With continual advancements in payload and sensor capabilities, we expect policies will be modified to further the use for UAS for agricultural applications; however, most research/breeding programs do not face this constraint. We advocate for greater involvement of practitioners to enable appropriate framing of policy.

We conclude by emphasizing that UAS systems are a very versatile and powerful approach for high-throughput phenotyping. While challenges remain, current developments suggest that the future is very promising for deployment of these systems for a diverse array of plant phenotyping tasks.

## Figures and Tables

**Table 1:** Brief description of types of UAV and their feature specifications.

	Payload (kg)	Flight time (minutes)	Operability	Price range	Ability to hover
Single-rotor (helicopter)	16-32	50-100	Difficult	High (for sprayer drones)	Yes
Multirotor	0.8-6	25-35	Easy	Low-high	Yes
Fixed-wing	<0.5	50-90	Medium	Mid-high	No
VTOL fixed-wing	<0.8	60	Medium	High	Yes (for takeoff)

**Table 2:** Main sensor types mounted as UAS payloads.

	# of bands (commonly available)	Commonly covered spectrum	Cost	Weight	Resolution (megapixel)	Ease of use
RGB	3	450-750 nm	Low	Low-medium	Low-high	Easy
Multispectral	3-10	450-1000 nm	Medium	Low-medium	Medium	Medium
Hyperspectral	>10	450-1000 nm	High	High	Low	Difficult
Thermal	1	3500-7500 nm	Medium	Low	Low	Medium
LiDAR	1^∗∗^	905 nm	Medium-high	Medium-high	Medium-high^∗^	Difficult

^∗^LiDAR resolution is not in megapixels but in point cloud density. ^∗∗^There are some multiband LiDAR systems, but they are not routine for UAS.

**Table 3:** Examples of software tools available for UAS way pointing.

Software name	Supported UAS	Manufacturer or 3rd party	Cost	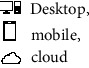	Note	Mapping function integrated	Website
Aerobotics flight planner tower	Autopilot board	3rd party	Free	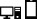	Dev is not active now. Works for Pixhawk series	No	[83]
Altizure	DJI	3rd party	Free	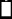	Provides 3D product visualization platform	Yes	[84]
Autopilot for DJI drones	DJI	3rd party	$	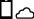	Provides flight recorder	No	[85]
DJI GS Pro	DJI	Manufacturer	Free	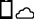	Needs to pay for additional functionalities	No	[86]
Drone Harmony Mission Planner	DJI	3rd party	$	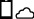	Provides full 3D intuitive interface	Yes	[87]
DroneDeploy	DJI	3rd party	Free	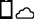	Needs to pay for additional function; provide live map	Yes	[88]
eMotion	senseFly	Manufacturer	$	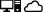	Needs basic knowledge of UAS to connect with UAS; need to work with the manufacturer UAS	No	[89]
Intel® Mission Control Software	Intel® Falcon™ 8+ UAS	Manufacturer	$	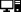	Needs basic knowledge of UAS to connect with UAS; functions only with the manufacturer of UAS	No	[90]
Litchi for DJI	DJI	3rd party	$	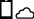	Needs additional mission planner	No	[91]
Map Pilot for DJI	DJI	3rd party	$	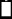	Needs to pay for additional functionality	Yes	[92]
mdCockpit app	Microdrones	Manufacturer	Free	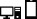	Needs basic knowledge of UAS to connect with UAS; functions only with manufacturer UAS	No	[93]
Mission Planner	Autopilot board	3rd party	Free	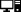	Needs basic knowledge of autopilot board, specifically (i.e., Pixhawk series) with Ardupilot or Px4 (or any other autopilot that communicates using the MAVLink protocol)	No	[94]
Pix4Dcapture	DJI; Parrot; Yuneec	3rd party	Free	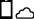	Supports upload to Pix4d cloud	Yes	[95]
QGroundControl	Autopilot board	3rd party	Free	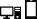	Needs basic knowledge of autopilot board (i.e., Pixhawk series) with Ardupilot or Px4 (or any other autopilot that communicates using the MAVLink protocol)	No	[96]
UgCS	DJI; autopilot board	3rd party	$	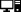	Needs basic knowledge of UAS to connect with UAS (i.e., Pixhawk series) with Ardupilot or Px4; Yuneec; MikroKopter; MicroPilot; Microdrones; Lockheed Martin	Yes	[97]

**Table 4:** Examples of software for analyzing and working with UAS data, including orthomosaicing, photogrammetry, and spectral index (e.g., NDVI) generation. The list is nonexhaustive.

Software	Parent	Commercial vs. open	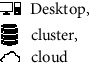	Website
3D Zephyr	3D Flow	$	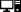	[127]
Drone2Map	ESRI Inc.	$	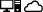	[128]
DroneDeploy	DroneDeploy Inc.	$	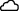	[129]
Farmers Edge	Farmers Edge Inc.	$	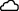	[130]
FlytBase	FlytBase Inc.	$	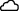	[131]
Metashape	Agisoft LLC	$	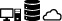	[132]
OneDroneCloud	Terra Imaging LLC	$	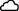	[133]
OpenAerialMap	Community	ᴒ	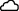	[134]
OpenDroneMap	Community	ᴒ	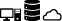	[124]
OpenSfM	Community	ᴒ	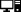	[135]
Pix4D	Pix4D Inc.	$	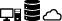	[136]
PrecisionMapper	PrecisionHawk	$	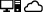	[137]
Remote Expert	DroneMapper	$	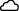	[138]
Skycatch		$	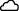	[139]

**Table 5:** Examples of the use of UAS for field phenotyping using the criteria of identification, classification, quantification, and prediction (ICQP) of traits. This is a nonexhaustive list.

ICQP	Type of plant trait	UAV type	Flight altitude (m)	Image resolution	Plant species	Plant trait analysis/model	Sensor on UAV	Plant phenotype	Ref.
Classification	Morphological and physiological	Multirotor	30	-	Vineyard	ANN	Multispectral sensor	Stem water potential, water stress	[172]
Quantification	Physiological	Multi rotor	50	~2.2 cm and 1.11	Winter wheat	ANN, SVM, RF, BBRT, DT, MLR, PLSR, and PCR	Hyperspectral and RGB	Aboveground biomass (AGB)	[173]
Quantification	Physiological	Multirotor & fixed-wing	40	-	Forest, soybean, Sorghum	ANOVA, correlation and heritability	Thermal imaging	Water stress	[58]
Quantification	Physiological	Multirotor	80	1.51 cm per pixel	Maize	Broad-sense heritability and genetic correlations	RGB	Crop cover and senescence	[174]
Quantification	Physiological	Multirotor	30	0.5 cm	Potato	Correlation, RF	RGB	Crop emergence	[175]
Identification	Morphological trait	Multirotor	75	5 cm/pixel	Citrus trees	DCNN	Multispectral	Counting trees	[176]
Quantification	Morphological	Multirotor	40 and 50	13 and 10 mm/pixel	Sorghum	Genomic prediction	RGB or near-infrared green and blue (NIR-GB)	Plant height	[27]
Quantification	Physiological, abiotic stress	Multirotor	50, 120	7.2, 3 cm/pixel	Dry beans	GNDVI, correlation	Multispectral	Seed yield, biomass, flowering, drought	[177]
Classification and quantification	Physiological	Multirotor	25	1.5–3.5 cm per pixel	Wheat	Heritability, correlation and GWAS	RGB and multispectral	Lodging	[178]
Quantification	Morphological and physiological trait	Multirotor	50	2.16 × 2.43 cm (snapshot), 1.11 × 1.11 cm (digital)	Wheat	Linear regression, RF, PLSR	RGB, spectroradiometer, and snapshot hyperspectral sensor	Crop height, LAI, biomass	[179]
Quantification	Physiological	Multirotor	30, 40	2.5, 2.8 cm	Bread wheat	Linear regressions, correlation matrix, and broad sense heritability	Multispectral	Senescence	[180]
Quantification	Physiological	Multirotor	75	5 cm/pixel	Cotton	Mixed linear model	Multispectral	Crop WUE	[181]
Quantification	Physiological	Multirotor	50	-	Maize	Multitemporal modelling	3D imaging and RGB	AGB	[182]
Quantification	Biotic stress	Multirotor	-	0.8cm	Potato	Multilayer perceptron and CNN	RGB and multispectral	Late blight severity	[183]
Quantification	Morphological	Multirotor	3-8	-	Blueberry bush	Multivariate analysis	RGB	Height, extents, canopy area and volume canopy width, and diameter	[184]
Quantification	Biotic stress	Multirotor	5.5, 27	-	Rice	NDVI and correlation	RGB and multispectral	Sheath blight	[185]
Quantification	Abiotic stress	Multirotor	13	0.5 and 1.12 cm	Tomato	OBIA	RGB and multispectral	Salinity stress plant area	[186]
Quantification	Biotic stress	Multirotor	15	0.6 cm	Cotton	OBIA	RGB	Cotton boll	[187]
Identification	Biotic stress	Multirotor	30, 60	0.01-0.03 m/pixel	Sunflower	OBIA	RGB, multispectral	Weed	[188]
Quantification	Physiological and morphological	Multirotor	20	6-8 mm	Eggplant, tomato, cabbage	RF and support vector regression	RGB images	Crop height, biomass	[189]
Classification	Biotic stress	Fixed	150	0.08 m/pixel	Vineyard	Receiver operator characteristic analysis	Multispectral	Flavescens dorée, grapevine trunk diseases	[190]
Quantification	Morphological	Fixed-wing	>100	2.5, 5, 10, 20 cm	Maize	Regression	RGB	Height	[80]
Quantification	Morphological	Multirotor	50, 29, 13	0.01 m	Cotton	Regression	RGB	Height	[191]
Quantification	Morphological	Multirotor	52.5	1.13 cm/pixel	Maize	Regression	RGB	Plant height	[192]
Quantification	Physiological	Multirotor	35, 70, 100	0.54, 1.09, and 1.57 cm)	Barley	Regression analysis	RGB	Lodging severity, canopy height	[193]
Quantification	Physiological	Multirotor	7	6 mm	Wheat	Regression analysis	RGB	Seed emergence	[194]
Quantification	Morphological and physiological	Multirotor	-	-	Wheat	Regression analysis	RGB images	Canopy traits	[195]
Quantification	Morphological	Multirotor	30	2.5 cm/pixel	Bread wheat	Regression, QTL mapping, and genomic prediction	RGB camera and 4 monochrome sensors (NIR, red, green, and red-edge)	Plant height	[196]
Quantification	Morphological	Multirotor	25	-	Oilseed rape	RF, regression analysis	RGB and multispectral	Flower number	[197]
Identification	Biotic stress	Multirotor	1, 2, 4, 8, 16	-	Soybean	SVM, KNN	RGB	Foliar diseases	[198]
Quantification	Morphological	Multirotor	30, 50, 70	-	Lychee crop	Tree height, crown width, crown perimeter, and plant projective cover	Multispectral	Crop structural properties	[199]
Quantification	Physiological	Multirotor	40, 60	-	Maize	Univariate and multivariate logistic regression models	RGB and multispectral	Lodging	[200]
Quantification	Biotic stress	Multirotor	80	-	Beet	Univariate decision trees	Hyperspectral	Beet cyst nematode	[201]
Quantification	Biotic stress	Multirotor	-	-	Peanut	Vegetation index	Multispectral	Spot wilt	[202]
Quantification	Morphological and physiological traits	Multirotor	20	-	Cotton	Vegetation index, SVM	Multispectral	Plant height, canopy cover, vegetation index, and flower	[203]
Quantification	Physiological	Multirotor	150	8.2 cm	Wheat	Vegetative index	Multispectral	LAI	[204]
Identification	Biotic stress	Multirotor	~10	-	Radish	VGG-A, CNN	RGB	Fusarium wilt	[205]

## Abbreviations

AGB: Aboveground biomass
ANN: Artificial neural network
BBRT: Boosted binary regression tree
CNN: Convolutional neural network
DT: Decision tree
DCNN: Deep convolutional neural network
GWAS: Genome-wide association study
GNDVI: Green normalized difference vegetation index
KNN: *K*-nearest neighbor
LAI: Leaf area index
MLR: Multivariable linear regression
NDVI: Normalized difference vegetation index
OBIA: Object-based image analysis
PLSR: Partial least squares regression
PCR: Principal component regression
RF: Random forest
SVM: Support vector machine
VGG: Visual geometry group
WUE: Water use efficiency.
